# A Nanoporous 3D-Printed Scaffold for Local Antibiotic Delivery

**DOI:** 10.3390/mi15010083

**Published:** 2023-12-30

**Authors:** Pouyan Ahangar, Jialiang Li, Leslie S. Nkindi, Zohreh Mohammadrezaee, Megan E. Cooke, Paul A. Martineau, Michael H. Weber, Elie Saade, Nima Nateghi, Derek H. Rosenzweig

**Affiliations:** 1 Department of Surgery, McGill University, Montreal, QC H3G 1A4, Canada; pouyan.ahangar@mail.mcgill.ca (P.A.); megan.cooke@colorado.edu (M.E.C.); paul.martineau@mcgill.ca (P.A.M.); michael.weber@mcgill.ca (M.H.W.); 2 Department of Science, TAV College, Montreal, QC H3W 3E1, Canada; arthur1998lee@gmail.com (J.L.); lnkindi37@gmail.com (L.S.N.); zohrehmohammadrezaee@gmail.com (Z.M.); saade.elie@tav.ca (E.S.); nnateghi@tav.ca (N.N.); 3 Injury, Repair and Recovery Program, Research Institute of McGill University Health Centre, Montreal, QC H3G 1A4, Canada

**Keywords:** tissue engineering, drug delivery, antibiotics, bone defect, antimicrobial resistance

## Abstract

Limitations of bone defect reconstruction include poor bone healing and osteointegration with acrylic cements, lack of strength with bone putty/paste, and poor osteointegration. Tissue engineering aims to bridge these gaps through the use of bioactive implants. However, there is often a risk of infection and biofilm formation associated with orthopedic implants, which may develop anti-microbial resistance. To promote bone repair while also locally delivering therapeutics, 3D-printed implants serve as a suitable alternative. Soft, nanoporous 3D-printed filaments made from a thermoplastic polyurethane and polyvinyl alcohol blend, LAY-FOMM and LAY-FELT, have shown promise for drug delivery and orthopedic applications. Here, we compare 3D printability and sustained antibiotic release kinetics from two types of commercial 3D-printed porous filaments suitable for bone tissue engineering applications. We found that both LAY-FOMM and LAY-FELT could be consistently printed into scaffolds for drug delivery. Further, the materials could sustainably release Tetracycline over 3 days, independent of material type and infill geometry. The drug-loaded materials did not show any cytotoxicity when cultured with primary human fibroblasts. We conclude that both LAY-FOMM and LAY-FELT 3D-printed scaffolds are suitable devices for local antibiotic delivery applications, and they may have potential applications to prophylactically reduce infections in orthopedic reconstruction surgery.

## 1. Introduction

The management of critical-sized bone defects continues to be a challenging problem in orthopedics. Such defects are not capable of spontaneous healing without intervention due to their large size. Common causes of bone defects include high energy trauma, infection, and cancer [1]. Current surgical management techniques include bone grafting, distraction osteogenesis, and the induced membrane technique. Although these techniques have been shown to be effective in varying degrees in the literature, large-scale randomized controlled trials are lacking to determine the best treatment approach for large bone defects, mainly due to the low incidence of the condition [2]. Additionally, there are limitations attributed to each technique. Autologous bone grafts are quite effective in inducing bone growth due to their osteoconductive and osteoinductive properties. However, the limited available quantity in a patient, donor site morbidity, and resorption by the body make them unfeasible in large bone defects [2,3,4]. Similarly, allografts have limited effectiveness in inducing bone growth as a stand-alone solution. However, these two techniques and others may require multiple operations, increasing patient morbidity and risk of infection [3,5]. Distraction osteogenesis is effective but inefficient due to the very long treatment durations required and the high risk of infection at the pin sites for the distraction mechanism [2]. Although effective to varying degrees, none of these techniques address all the requirements for managing critical-sized bone defects effectively while minimizing surgical morbidity for patients and there remains a risk of infection at the reconstruction site and surgical wound [6,7].

Tissue engineering is a rapidly expanding field in regenerative medicine using cells, scaffold biomaterials, and biologic factors [8] which may provide unique solutions to managing critical-sized bone defects. Tissue engineering in bone regeneration has focused on developing bioactive implants with osteoconductive and osteoinductive properties. One advantage of tissue engineering is its ability to utilize 3D printing to create highly customizable implant structures to achieve the desired structural integrity and porosity for creating a suitable bone scaffold [2,9]. These scaffolds can be made from biocompatible porous materials coveted in tissue engineering applications [10]. They can subsequently be loaded with bioactive compounds such as growth modulators, chemotherapeutics, and antibiotics [11] to aid in the proliferation and differentiation of host cells for full osteointegration of the implant and subsequent healing of a bone defect [12]. This “all-in-one” solution can allow for implantation of a therapeutic-loaded structural implant at the site of a bone defect to restore structural integrity to the affected bone [3,5]. Subsequent elution of a cocktail of therapeutics, including prophylactic antibiotics, can promote osteointegration and prevent further infection, especially in high energy trauma scenarios. This approach would solve many of the limitations associated with current surgical options including a scarcity of autogenous bone graft and the need for multiple operations, as seen with the induced membrane technique.

The current clinical standard for local antibiotic delivery to bone defects is the use of antibiotic-impregnated polymethyl methacrylate (PMMA) cement, which is free-formed intraoperatively to fill the defect [13]. Powdered antibiotic is usually added to the cement composition during mixing intraoperatively, which can limit use of various antibiotics sensitive to the exothermic polymerization process [14]. Much of the antibiotic release has been attributed to a surface burst effect and increasing the concentration of antibiotics in the cement mixture leads to a weaker cement structure [13]. Alternate materials with higher surface to volume ratios may be useful for better controlled release in orthopedic implants. Additive manufacturing in the form of 3D printing has been increasingly used to generate custom bioresorbable implants with high mechanical strength.

Three-dimensional printing exists in several forms, with stereolithography, laser sintering, powder printing, fused-deposition modeling (FDM), and bioprinting to name a few [15]. Each type of printing has different processes for layering the prints, and each one can utilize different materials. Synthetic biocompatible polymers have been established as a highly versatile substrate for 3D printing such scaffolds using FDM technology. Our lab has previously demonstrated the 3D printability and osteoconductive properties of a commercial line of synthetic polymer (PORO-LAY), alongside its ability to sustainably release chemotherapeutics [16,17]. The PORO-LAY line of thermoplastic 3D-printed filaments are a novel commercial product composed of a rubber elastomeric polymer with a soluble polyvinyl alcohol (PVA) component [18,19]. They are hydrophobic in nature and act as a sponge-like structure, which can swell with water and diffuse molecules. These unique filaments can be 3D printed into a customized solid shape which upon washing away the PVA becomes a micro-nano porous structure suitable for drug uptake [19,20]. We and others have also demonstrated that this filament can be printed into custom geometries that are both cytocompatibile in vitro [21] and biocompatible in vivo [22]. The PORO-LAY line of filaments is available in different formulations that achieve different porosity characteristics, presumably by altering the ratio of PVA to the rubber component. Specifically, the LAY-FOMM variant transforms into a foam-like material, with micropores scattered throughout the polymer structure upon dissolution of the soluble PVA component. Alternatively, the LAY-FELT variant transforms into a felt-like material with woven strands of the polymer. These materials are not only suitable for drug delivery at implant sites, but may also be applicable to surgical site wound infections, which are not uncommon following orthopedic surgery [23]. Therefore, loading of such materials with antimicrobial agents may facilitate prophylaxis against infection; however, it remains to be determined. Furthermore, our and others’ previous studies have suggested prophylactic antibiotic delivery may prevent such infections from occurring altogether [24,25]. Our goal in this study is to compare the drug release rate of antibiotics between the foam and felt variants of this filament in an in vitro bacterial culture model. We also compare the impact of various infill patterns. The results of this study can help demonstrate the effectiveness of this approach for targeted antibiotic delivery in the design of future tissue-engineered implants for managing critical-sized bone defects or surgical site wound infections.

## 2. Materials and Methods

### 2.1. Three-Dimensional Printing of Disks

**Figure 1:**
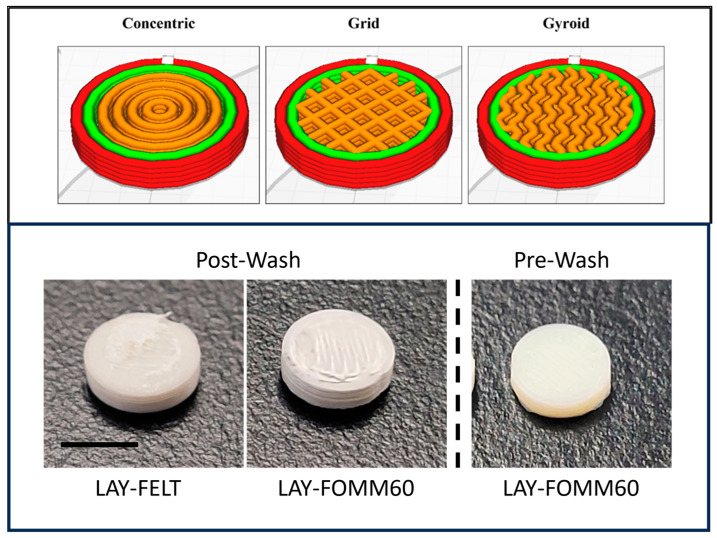
LAY-FOMM and LAY-FELT scaffolds were printed first in larger format to characterize the print consistency with concentric, grid, and gyroid infill patterns, without solid top or bottom. The dimensions were then switched to 6 mm, 0.8 mm discs for antibiotic release to make them comparable to the commercial paper disks. Bottom images are representative LAY-FELT and LAY-FOMM60 printed scaffolds after washing for 72 h. The far right image shows a pre-washed LAY-FOMM60 scaffold to show how much they swell after washing. Scale bar is 5 mm.

Printing filaments were purchased from Matterhackers (Burbank, CA, USA). The two filament types were the PORO-LAY LAY-FOMM 60 porous filament 1.75 mm (Product number: M-LWK-N3PY) and PORO-LAY LAY-FELT porous filament 1.75 mm (Product number: M-8V4-0Q73). The disks were 3D modeled with a 6 mm diameter and 1 mm depth and sliced into G-code using the Simplify3D software V 4.1.2 (Simplify3D, Cincinnati, OH, USA). Batches with 3 different infill patterns (Figure 1) were then printed (concentric, gyroid, grid) using a Flashforge Creator Pro (Flashforge Corp., Jinhua, Zhejiang, China), with a 0.3 mm nozzle, print settings of 220 °C print temperature, 50 °C bed temperature, and 18 mm/s speed. Separate batches were printed with Layfomm and Layfelt. The diameter and height of each disk were measured using a micrometer, as well disk weight using a calibrated microgram scale.

### 2.2. Scanning Electron Microscopy

Scanning electron microscopy on the 3D-printed scaffolds was performed exactly as described elsewhere [22]. In short, printed scaffolds (both LAY-FOMM and LAY-FELT, with concentric, gyroid, and grid infill patterns) were dehydrated using increasing concentrations of ethanol up to 100% and then overnight in hexamethyldisilazane (HDMS, Sigma, Oakville, ON, Canada). All samples were sputter-coated with 4 nm layer of platinum using an ACE600 sputter-coater (Leica, Burlington, ON, Canada). Images were captured using an FEI Quanta 450 Scanning Electron Microscope (ThermoFisher, Burlington, ON, Canada).

### 2.3. Antibiotic Solution Preparation

In total, 0.03 g of Tetracycline powder was mixed in 4 mL of solution inside a 50 mL beaker. The solution was transferred to a clean 15 mL tube and allowed to settle. To remove any undissolved solutes, ¾ of the solution was transferred to a clean beaker and allowed to settle. The process of transferring the solution from a beaker to a 15 mL tube was repeated once more to remove any remaining undissolved solutes. The 15 mL tube containing the final solution was mixed for 3 min using a mixer.

### 2.4. Disk Sterilization and Loading

Three-dimensional printed disks were soaked in distilled water for 3 days with 24 h water changes. They were next placed in 70% isopropanol for 5 min and then washed in sterile water to dispose of extra alcohol on the disks. Next, the disks were exposed to UV light for 10 min on each side. Disinfected (UV) Kimwipes were used to remove extra water over the disks. After, 5 μL of antibiotic solution was deposited onto each disk, and they were allowed to dry in the biosafety cabinet for 30 min before transferring to the agar plate. The commercially acquired antibiotic or blank disk was used directly from the package under sterile conditions.

### 2.5. Agar Plate Preparation

*Staphylococcus aureus* (cat# 470179-208) and *Pseudomonas fragi* (cat# 470179-090) cultures (VWR, Mississauga, ON, Canada) were used in this study. To subculture the two organisms, single colonies were swabbed from each plate and streaked as new cultures on agar plates made with tryptic soy broth. Individual colonies were suspended in 1 mL sterile PBS and agitated for 2 h at 37 °C to activate the culture. Fresh agar plates were labelled into 8 different zones (H_2_O, N and 6 different disk zones). The 1 mL culture was then plated on the agar plate and spread evenly (Figure 1). Disks were placed as indicated and the plates were placed in 37 °C incubator for the indicated times.

### 2.6. Inhibition Zone Test Procedure

**Figure 2:**
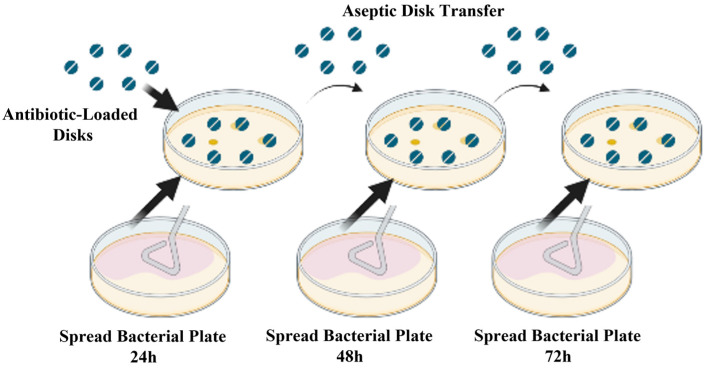
Schematic view of inhibition zone test and disk transfer procedure over the 72 h experiment. Antibiotic-loaded control disks and 3D-printed disks are placed on the bacterial spread plate and incubated for 24 h, and diameter of growth inhibition zone is measured. The disks are aseptically transferred to the next bacterial spread and the procedure is repeated two more times to measure the sustained release of antibiotics from the disks.

For controls, a Whatman paper and a 3D-printed scaffold (concentric infill) not loaded with antibiotic were used. The remaining 4 disk zones were deposited with tetracycline-loaded 3D-printed scaffolds with 3 different infills and a commercial disk. The disk-loaded plates were incubated for 24 h, and then transferred to another plate and placed in the incubator for 24 h (total of 48 h) and transferred once more, for a total of 72 h (Figure 2).

### 2.7. Metabolic Activity

Three-dimensional printed disks using LAY-FOMM or LAY-FELT were sterilized as outlined before and placed inside a 24-well plate. Some disks were loaded with antibiotics as outlined before and some were left blank. Anterior cruciate ligament (ACL) fibroblasts were isolated from the samples collected from 3 patients with consent and approved by RI MUHC ethics REB #2019-5390, as previously described [26]. The fibroblasts were suspended in culture media at a concentration of 250,000 cells/well were cultured on the disks for 24 h in an incubator at 37 °C. Next, cell metabolic assay using a commercial Alamar Blue^®^ kit (ThermoFisher, Burlington, ON, Canada) was conducted according to manufacturer instructions into each well. After 24 h, 10 μL of Alamar Blue^®^-containing culture media was plated in a 96-well plate (Costar, Sigma, Oakville, ON, Canada) and fluorescence (Excitation–540 nm, Emission–585 nm) was analyzed using the Tecan Infinite M200 pro microplate reader (Tecan Trading, AG, Männedorf, Switzerland). Three wells containing culture media and Alamar Blue without any scaffold or cells were also read on the same plate to represent background fluorescence of the Alamar Blue dye. The average fluorescence from these wells was subtracted from the experiment fluorescence readings to remove background fluorescence. Results were analyzed using Microsoft Excel and *t*-test analysis was conducted.

### 2.8. Live/Dead Cell Viability Assay

Fibroblast cells were cultured onto disks according to the protocol in the metabolic activity section. After 24 h incubation, live/dead assay (ThermoFisher, Burlington, ON, Canada) was performed according to manufacturer instructions. Plates containing live/dead assay were imaged using an Olympus IX73 inverted fluorescence microscope. Image J software (NIH, v.1.6.0, Bethesda, MD, USA) was used to quantify the live and dead cells. Results were analyzed using Microsoft Excel and *t*-test analysis was conducted.

## 3. Results

### 3.1. Filament Surface Analysis and Print Quality Control

**Figure 3:**
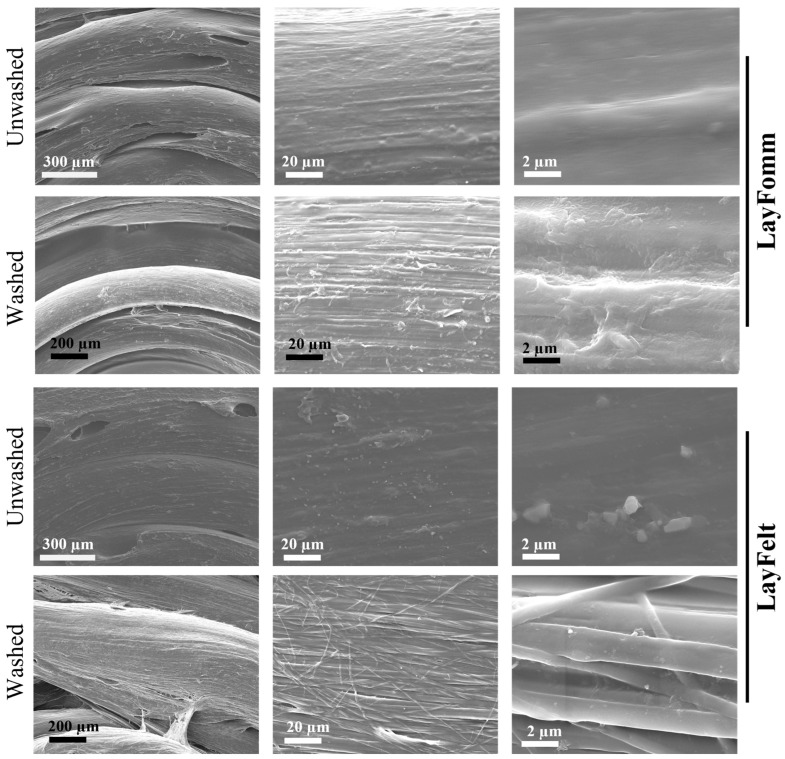
Scanning electron microscopy images showing the different surface texture of LAY-FOMM and LAY-FELT disks after being washed.

We investigated microscopic changes in the surface structure of both 3D-printed filaments before and after the soluble PVA component was dissolved in a water bath (Figure 3). Visually, both filaments demonstrated a smooth surface at higher magnification in the unwashed state. Upon washing and dissolution of the soluble component, different surface changes were noted in each filament at a higher magnification. In particular, LAY-FOMM demonstrated more pronounced parallel fibers at 20 µm and a roughened pitted texture at 2 µm with no specific polymer strands visible. In contrast, LAY-FELT demonstrated a more pronounced weave pattern of fibers at 20 µm with individual strands visible at 2 µm.

**Figure 4:**
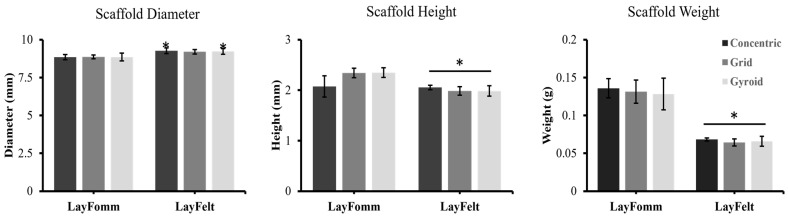
Scaffold weights, heights, and diameters were measured immediately after printing. LAY-FOMM and LAY-FELT scaffolds with concentric, grid, and gyroid patters were measured. Scanning electron microscopy images showing the different surface texture of LAY-FOMM and LAY-FELT disks after being washed. Scaffolds were printed larger for characterization and print consistency testing, and then switched to 6 mm, 0.8 mm discs for antibiotic. All error bars = SD, with a minimum of n = 13 for each infill pattern, * indicates *p* less than 0.001, two-tailed *t*-test for each group comparison.

To ensure each printed material and infill pattern resulted in consistent output from the low-cost desktop 3D printers, individual weights, diameters, and heights were measured for each print. A minimum number of 13 scaffolds was printed in each infill pattern for each filament type. As for disk diameter, we noted a statistically significant lower print diameter in all three infill patterns of the LAY-FOMM disks compared to LAY-FELT (concentric (mm) = 8.86 vs. 9.27; grid (mm) = 8.87 vs. 9.21; gyroid (mm) = 8.86 (Figure 4). As for disk height, there was no difference in height between the two filament types with a concentric infill (2.08 mm vs. 2.05 mm; *p* 0.79). However, LAY-FOMM had a statistically significant increased height compared to LAY-FELT (grid (mm) = 2.34 vs. 1.99; gyroid (mm) = 2.35 vs. 1.98; *p* < 0.001). Lastly, the LAY-FOMM disks were on average twice as heavy as the LAY-FELT filaments, regardless of the infill pattern (concentric (g) = 0.14 vs. 0.068; grid (g) = 0.13 vs. 0.064; gyroid (g) = 0.13 vs. 0.066; *p* < 0.001).

### 3.2. Inhibition Zone Test

**Figure 5:**
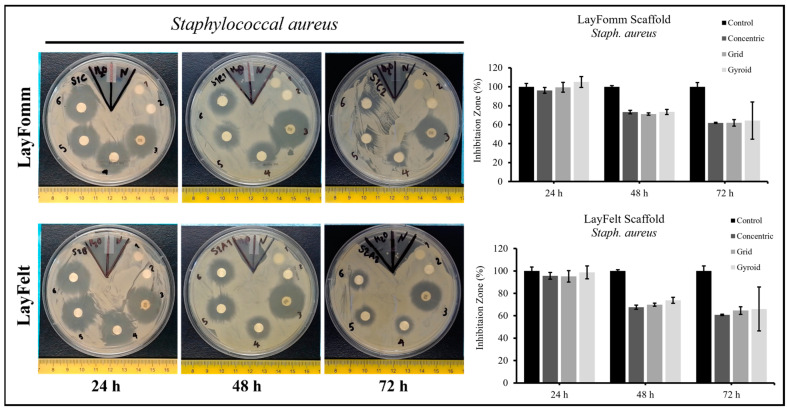
Tetracycline loaded on 6 mm, 1 mm discs for antibiotic release, 1 = control Whatman paper, 2= control 3D scaffold, 3 = commercial disc, 4 = concentric, 5 = gyroid, 6 = grid. Error bars = SD, n = 3.

**Figure 6:**
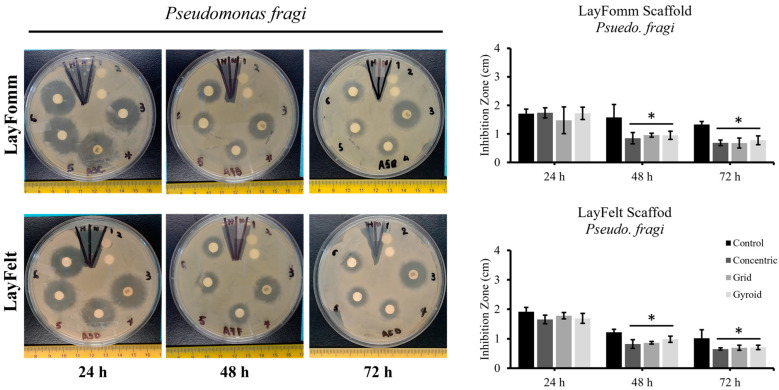
Tetracycline loaded on 6 mm, 0.8 mm discs for antibiotic release, 1 = control Whatman paper, 2 = control 3D scaffold, 3 = commercial disc, 4 = concentric, 5 = grid, 6 = gyroid. Error bars = SD, n = 5. * indicates *p* < 0.05.

**Figure 7:**
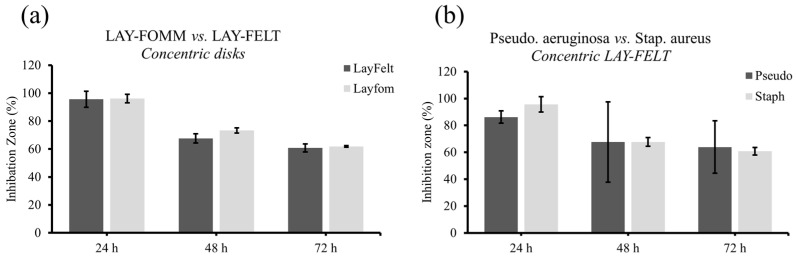
Comparison of inhibition zones (%) of (**a**) LAY-FELT and LAY-FOMM for *Staph. aureus* and (**b**) concentric LAY-FELT disks for both bacteria cultures.

The 3D-printedfilaments used in this study have the unique ability to transform into a porous structure upon dissolution of their soluble component, making them suitable for absorbing and releasing therapeutics. Both filaments tested in this study achieve this in different manners, with the LAY-FOMM filament forming a foam-like structure with micropores, while the LAY-FELT filament forms a felt-like filamentous structure. Here, we set out to test the difference in sustained antibiotic release between these two filaments as well as with different 3D-printed infill patterns. We utilized a commercially available antibiotic disk as a control. The 3D-printed and control disks were loaded with the antibiotic Tetracycline as outlined in Section 2. Antibiotic-loaded disks were placed in agar plates cultured with *Staph. aureus* (Figure 5) or *Pseudomonas fragi* (Figure 6) and inhibition zones were measured at 24 h, 48 h, and 72 h time points. A total of three replicates were conducted for *Staph. aureus* and five replicates for *Pseudomonas fragi* dishes. Overall, we observed that 3D-printed scaffolds achieved a lesser degree of sustained antibiotic release compared to commercial disks, reflected through a smaller inhibition zone for longer incubation periods. Table 1 and Table 2 report the inhibition zone (%) as compared to the control disk for both bacteria cultures for the LAY-FELT and LAY-FOMM disks, respectively. Comparing the % zone of inhibition of the 3D-printed disks compared to the control disks, we observed no significant difference in the inhibition zones of the LAY-FOMM and LAY-FELT disks for either of the bacteria cultures (*p* ≈ 0.5 from two tailed *t*-test), as shown in Figure 7a,b.

### 3.3. Metabolic Activity and Cell Viability

**Figure 8:**
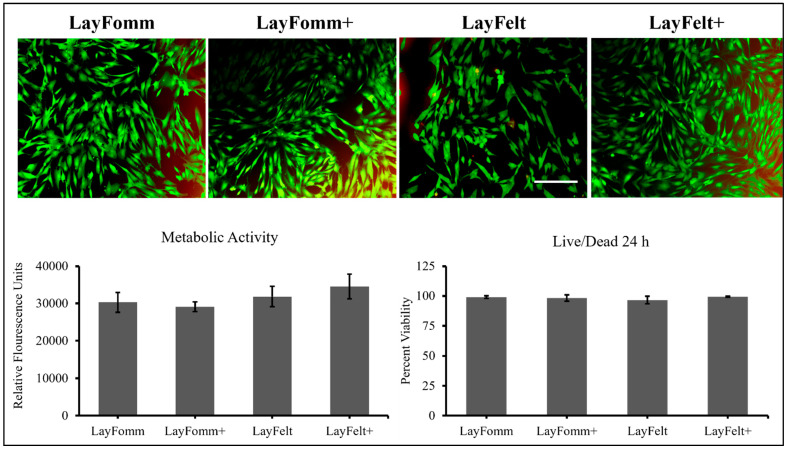
Tetracycline-loaded (+) or unloaded 6 mm, 0.8 mm discs (gyroid), cultured for 24 h with 250,000 primary human ACL fibroblasts. Alamar Blue assay was performed to measure metabolic activity and cellular health/toxicity followed by LIVE/DEAD assay, whereby dead cells appear red and live cells appear green. Data shown from 3 independent ACL donors, ±SD.

To determine whether disks loaded with or without antibiotics showed any cytotoxicity, we tested the materials in culture with primary human fibroblasts. Alamar Blue cell metabolic activity and live/dead assay (Figure 8) showed that the fibroblast cells maintained metabolic activity (LAY-FOMM no antibiotic vs. antibiotic 29,127.67 units vs. 29,127.67 units (*p* = 0.27); LAY-FELT no antibiotic vs. antibiotic 31,857.67 units vs. 34,550.67 units (*p* = 0.17)) with no increase in the amount of cell death (LAY-FOMM no antibiotic vs. antibiotic 99% vs. 98% (*p* = 0.70); LAY-FELT no antibiotic vs. antibiotic 97% vs. 99% (*p* = 0.27)) when cultured on the scaffold material. There was no statistical difference between filament types and whether antibiotics were loaded on the scaffolds or not. Regarding live/dead assay, we noted negligible cell death after 24 h and cell viability remained at more than 96%.

## 4. Discussion

We have recently shown that LAY-FOMM, a commercial polyurethane/polyvinyl alcohol blend thermoplastic, has sustainable release properties for the chemotherapeutic doxorubicin [16] and the bisphosphonate zolodronate [17]. Furthermore, these drug-loaded scaffolds can be used to effectively block the growth of patient-derived spine metastases cells in vitro. We have now tested a scaffold printed from another commercial PORO-LAY nanoporous 3D-printed filament, LAY-FELT, which shows different properties at the nanoscale (Figure 3). Both materials are blended with polyvinyl alcohol, which dissolves in water or buffer to expose tiny holes in the nanoscale range and resemble sponge-like and elastic properties [27,28]. Furthermore, the visual appearance of the LAY-FELT scaffolds resembles aligned collagen or extracellular matrix fibrils, which may be even more conducive to tissue repair or drug delivery applications. Our results here indicate compatibility with the uptake and sustainable release of antimicrobials. The data also indicated no negative effects on primary human fibroblasts cultured with scaffolds alone or loaded with antibiotics (Figure 8). Our most recent work with LAY-FOMM has shown that it is cytocompatible with human osteoblasts, can promote stem cell osteogenic differentiation, is biocompatible in vivo, and can promote bone formation in mandible defects [22]. Together, these data suggest that such materials may be good candidates for sustained local antibiotic delivery as well at topical or implanted sites.

Our results indeed demonstrate the capacity of both the LAY-FOMM and LAY-FELT materials in the extended release of antibiotics, which is suitable for delivering antibiotics locally/prophylactically while promoting tissue repair in orthopedic surgical sites [22]. We tested three infill patterns corresponding to different surface areas, with the concentric pattern having the least surface exposed to the antibiotic while being loaded and the gyroid pattern having the most. The infill pattern had no significant impact on the release rates, as observed in Figure 4 and Figure 5 (*p* > 0.4 from a two tailed *t*-test). This may correspond to the fact that although the concentric pattern has the least surface to absorb the antibiotics, it has the largest mass compared to the other two infill patterns as the disks were printed with the same diameters and heights. Hence, we were not able to quantify the impact of the surface on releasing the antibiotic. We plan to systematically study the effect of the surface by printing disks of the same infill pattern and volume but different surface areas (different surface to volume ratios). No significant difference in the inhibition zone of the LAY-FOMM and LAY-FELT disks (*p* > 0.4) was observed either, which indicates that the release mechanism of the antibiotic is the same for both material systems, within the range of our experimental parameters. Figure 7a shows an example of such comparison for the concentric disk. The other infill patterns showed the same trend. Additionally, we did not assess the impact of infill patterning on the mechanical strength of these scaffolds. However, our previous work [22,27] showed that LAY-FOMM60 is quite elastic with significantly lower mechanical strength than PLA, which is in agreement with other mechanical testing of these materials [29,30,31]. Even though this material was able to promote bone repair in a mandible defect, this would have to be considered for load bearing applications and regeneration in large bone defects.

There have been many studies over the past decades investigating antibiotic release from various polymers or mixes [32,33]. The polymers or blends often included the use of ceramics or thermoplastics such as polylactic acid and polycaprolactone. Indeed, commercially available antimicrobial products available for orthopedic applications are sold by Johnson & Johnson, Stryker, or Heraeus, such as SMARTSET bone cement with Gentamycin preloaded [34] or Smartset or Palacos cements with Tobramycin or Vancomycin [35]. Our focus in this study was the use of porous polyurethane-based scaffolds for antibiotic delivery. In the past, polyurethane coatings have been applied to implants to facilitate antibiotic delivery with some success [36,37,38]. A composite bioink consisting of polyurethane and polyethylene–glycol was also 3D printed into small structures [39] for antibiotic release and was successfully tested in vitro. Our study goes steps further by fabricating the entire polyurethane scaffold on its own using additive manufacturing technology, which allows for anatomic geometries towards implant design [16,17,22,40]. Furthermore, we showed that changing the infill patterns of the scaffolds may have an influence on the diffusion capacity. Others have shown that 3D-printed ceramics can be used for antibiotic delivery in bone implants [41], which is not too far removed from the commercially available bone cements which may be of preference to orthopedic surgeons. Another recent use of 3D printing for antibiotic release was in the form of polymethyl methacrylate microbeads incorporated into PCL thermoplastics. In that study [42], the rifampicin antibiotic was able to be printed at temperatures around 60 °C without losing its effective properties. Here, we avoided any potential heat-induced complications by first printing our scaffolds and then loading exact amounts of antibiotics. This opens up the possibility of using several therapeutics which are sensitive to temperatures above 37 °C.

The commercial impact of the PORO-LAY series of 3D-printed filaments has been explored over the past 5 years. Until now, LAY-FOMM and LAY-FELT 3D-printed scaffolds have been assessed as low-cost filtration [43,44,45] or molecular collection devices [28,46,47]. In all cases, these studies show how well these low-cost 3D-printed materials can capture small molecules through intricately designed scaffolds or patterns. Interestingly, the common theme among these studies is that they all focus on the uptake and molecular capture abilities of the PORO-LAY materials. To the best of our knowledge, our group has been the only one to study these materials in the context of tissue regeneration [22,26,27] and molecular uptake and sustained release [16,17]. Hence, these 3D-printed scaffolds are not only useful in an industrial/commercial setting but also in a clinical/translational setting. In terms of biocompatibility, we showed here that the drug-loaded scaffolds do not impact the viability of primary human fibroblasts. This is in alignment with our previous in vitro cytocompatibility tests with primary human osteoblasts [22] and ligament fibroblasts [26], as well as in vivo bone defect implantation of LAY-FOMM 60 [22]. Future work will test antibiotic release using sophisticated biofabricated human skin and bone infection models. This will then be followed by in vivo orthopedic and topical animal infection studies as well to determine clinical translational feasibility.

Our results showed that during the first 24 h, regardless of their material or infill pattern, all the disks have a similar drug release as compared to the control Whatman disk. The LAY-FOMM and LAY-FELT disks also showed a similar release rate through 48 h and 72 h, which was significantly lower than that of the control disk (*p* < 0.05). Similar release rates during the first 24 h suggest that the diffusion mechanism is the same for all the disks. Moreover, they suggest that the nature of bonding between the disk material and the antibiotic is the same for both 3D-printed and control disks. Although, FTIR measurements are necessary to confirm the nature of interaction (chemical vs. physical bonding). Lower antibiotic release rates from the 3D-printed disks during the next 24 h (total 48 h) and 48 h (total 72 h) suggest that the bulk diffusion through the 3D-printed disks is slower than that of the Whatman paper (control disk). Since less antibiotics were released from the 3D-printed disks over a 72 h period, we would anticipate a sustained or prolonged release of the drug, which is advantageous for applications where extended release is desired. To further confirm this hypothesis, we plan to extend our observation time until all of the loaded antibiotic is released from the disks.

Our goal in this study was to test the feasibility of using 3D-printed LAY-FOMM and LAY-FELT devices for antibiotic uptake and release. However, there are several important factors and concerns which must be raised concerning moving a device toward the market and clinical applications. Firstly, on the translational side of work to be conducted with our 3D-printed devices, in vivo topical applications as well as implant applications must be performed using small and large animals. This would provide important feedback on the biocompatibility and potential risks of device rejection. Second, the mechanical stability of the device (both shelf life and application stability) must be considered since we did not perform any long-term analysis of degradation in vitro or in vivo. Third, good manufacturing procedures and standards would have to be implemented for a workflow of the device’s manufacture, loading of antibiotics, sterilization, packaging, and delivery to clinics/operating rooms. This is no small feat and must be carefully orchestrated. Lastly, cost considerations much be taken into account, along with health and safety for patients, since the patient will likely have to consent to using such a device and pay for it through their insurance provider. All these caveats need to be considered when moving such a 3D-printed biomedical device toward the market and clinic, and they have been considered by others in great detail [10,15].

One major limitation of our study is that the release of antibiotics from each 3D-printed scaffold was only monitored over 3 days. To determine whether this release can be sustained past the initial burst effect (24 h), experiments over 1–4 weeks should be conducted, similar to our past studies [16,17]. Another limitation in our study was the use of simplified bacterial cultures on agar plates. A more robust series of experiments could be conducted with our drug eluting scaffolds on either mammalian cell/infection cultures [48] or even more sophisticated 3D-bioprinted or organotypic infection models [49,50]. Two common microbes found in orthopedic infections or wound closure infections were used in this study; yet, more species of microbes could certainly be tested. As such, we only examined the release of Tetracycline from our scaffolds as proof of concept. Therefore, a panel of various antibiotics and antimicrobial agents should be tested as well [51] such as amoxicillin with clavulanic acid or vancomycin. Finally, the clinical translational relevance of our 3D-printed drug delivery devices needs to be shown for in vivo applications, such as rodent bone infection [52] or skin infection models [53]. Taken all together, future studies using our drug delivery devices may lead to novel approaches for treating antimicrobial-resistant infections or may even act prophylactically against biofilm formation.

In conclusion, the combined data gathered from this study show without a doubt that both LAY-FOMM60 and LAY-FELT 3D-printed scaffolds can take up and release a standard antibiotic, Tetracycline. The release of the antibiotic from a single scaffold was effective at blocking two species of bacterial growth for at least 3 days. The infill patterns which changed the surface area of the scaffolds did not affect the antibiotic release or efficacy. Future work should explore studying the uptake and release of a panel of clinically relevant antibiotics, as well as a panel of clinical patient-derived bacteria. Also, since these types of scaffolds have been previously shown to be both cytocompatible and biocompatible, future studies demonstrating their efficacy against bacterial infections in vivo are warranted.

## Figures and Tables

**Table 1:** Inhibition zone (%) for the LAY-FELT disks relative to the control, ± SD.

**Bacteria**	**Disks**	**24 h**	**48 h**	**72 h**
*Staph. aureus*	Control	100 ± 1.9	100 ± 2.9	100 ± 1.9
	Concentric	95.6 ± 5.7	67.7 ± 3.3	60.8 ± 2.9
	Grid	95.2 ± 4.3	69.9 ± 6.0	64.6 ± 2.7
	Gyroid	98.8 ± 1.8	73.8 ± 1.9	66.1 ± 2.6
*Psuedo. fragi*	Control	100 ± 7.7	100 ± 8.4	100 ± 27.9
	Concentric	91.1 ± 4.6	76.9 ± 29.8	68.7 ± 20.0
	Grid	96.9 ± 5.7	77.3 ± 18.3	71.4 ± 18.6
	Gyroid	92.9 ± 9.0	85.4 ± 17.8	72.0 ± 14.3

**Table 2:** Inhibition zone (%) for the LAY-FOMM disks relative to the control, ± SD.

**Bacteria**	**Disks**	**24 h**	**48 h**	**72 h**
*Staph. aureus*	Control	100 ± 3.6	100 ± 1.2	100 ± 4.5
	Concentric	96.2 ± 3.0	73.3 ± 1.9	61.98 ± 0.6
	Grid	99.5 ± 5.1	71.2 ± 1.3	62.0 ± 3.5
	Gyroid	105.1 ± 5.8	73.5 ± 2.7	64.3 ± 19.7
*Pseudo. fragi*	Control	100 ± 9.0	100 ± 28.9	100 ± 7.9
	Concentric	101.5 ± 11.9	54.1 ± 12.8	51.4 ± 8.5
	Grid	86.4 ± 25.5	60.6 ± 17.3	51.1 ± 16.2
	Gyroid	100.7 ± 6.8	60.3 ± 12.1	58.4 ± 9.3

## Data Availability

All data presented in this study is contained within the study.

## References

[1] Roddy E., DeBaun M.R., Daoud-Gray A., Yang Y.P., Gardner M.J. (2017). Treatment of critical-sized bone defects: Clinical and tissue engineering perspectives. Eur. J. Orthop. Surg. Traumatol..

[2] Vidal L., Kampleitner C., Brennan M.Á., Hoornaert A., Layrolle P. (2020). Reconstruction of Large Skeletal Defects: Current Clinical Therapeutic Strategies and Future Directions Using 3D Printing. Front. Bioeng. Biotechnol..

[3] Ahangar P., Cooke M.E., Weber M.H., Rosenzweig D.H. (2019). Current Biomedical Applications of 3D Printing and Additive Manufacturing. Appl. Sci..

[4] Schmidt A.H. (2021). Autologous bone graft: Is it still the gold standard?. Injury.

[5] Ahangar P., Aziz M., Rosenzweig D.H., Weber M.H. (2019). Advances in personalized treatment of metastatic spine disease. Ann. Transl. Med..

[6] Masters E.A., Trombetta R.P., de Mesy Bentley K.L., Boyce B.F., Gill A.L., Gill S.R., Nishitani K., Ishikawa M., Morita Y., Ito H. (2019). Evolving concepts in bone infection: Redefining “biofilm”, “acute vs. chronic osteomyelitis”, “the immune proteome” and “local antibiotic therapy”. Bone Res..

[7] Fisichella L., Fenga D., Rosa M.A. (2014). Surgical Site Infection in Orthopaedic Surgery: Correlation between Age, Diabetes, Smoke and Surgical Risk. Folia Medica.

[8] Manzini B.M., Machado L.M.R., Noritomi P.Y., da Silva J.V.L. (2021). Advances in Bone tissue engineering: A fundamental review. J. Biosci..

[9] Kantaros A. (2022). 3D Printing in Regenerative Medicine: Technologies and Resources Utilized. Int. J. Mol. Sci..

[10] Kantaros A., Piromalis D. (2021). Fabricating Lattice Structures via 3D Printing: The Case of Porous Bio-Engineered Scaffolds. Appl. Mech..

[11] Visscher L.E., Dang H.P., Knackstedt M.A., Hutmacher D.W., Tran P.A. (2018). 3D printed Polycaprolactone scaffolds with dual macro-microporosity for applications in local delivery of antibiotics. Mater. Sci. Eng. C.

[12] Sallent I., Capella-Monsonís H., Procter P., Bozo I.Y., Deev R.V., Zubov D., Vasyliev R., Perale G., Pertici G., Baker J. (2020). The Few Who Made It: Commercially and Clinically Successful Innovative Bone Grafts. Front. Bioeng. Biotechnol..

[13] Steadman W., Chapman P.R., Schuetz M., Schmutz B., Trampuz A., Tetsworth K. (2023). Local Antibiotic Delivery Options in Prosthetic Joint Infection. Antibiotics.

[14] Wall V., Nguyen T.-H., Nguyen N., Tran P.A. (2021). Controlling Antibiotic Release from Polymethylmethacrylate Bone Cement. Biomedicines.

[15] Kantaros A. (2022). Bio-Inspired Materials: Exhibited Characteristics and Integration Degree in Bio-Printing Operations. Am. J. Eng. Appl. Sci..

[16] Ahangar P., Akoury E., Ramirez Garcia Luna A.S., Nour A., Weber M.H., Rosenzweig D.H. (2018). Nanoporous 3D-printed scaffolds for local doxorubicin delivery in bone metastases secondary to prostate cancer. Materials.

[17] Akoury E., Weber M.H., Rosenzweig D.H. (2019). 3D-Printed Nanoporous Scaffolds Impregnated with Zoledronate for the Treatment of Spinal Bone Metastases. MRS Adv..

[18] Cook T.S., Zhang J., Starosolski Z., Ezon D.S., Krishnamurthy R., Dodd N., Heinle J., McKenzie D.E., Annapragada A. Soft tissue models: Easy and inexpensive flexible 3D printing as a help in surgical planning of cardiovascular disorders. Proceedings of the Medical Imaging 2017: Imaging Informatics for Healthcare, Research, and Applications.

[19] Tsai K.J., Dixon S., Hale L.R., Darbyshire A., Martin D., de Mel A. (2017). Biomimetic heterogenous elastic tissue development. npj Regen. Med..

[20] Cui M., Chai Z., Lu Y., Zhu J., Chen J. (2023). Developments of polyurethane in biomedical applications: A review. Resour. Chem. Mater..

[21] Oberoi G., Nitsch S., Janjić K., Shokoohi-Tabrizi H., Moritz A., Moscato F., Unger E., Agis H. (2020). The impact of 3D-printed LAY-FOMM 40 and LAY-FOMM 60 on L929 cells and human oral fibroblasts. Clin. Oral Investig..

[22] Cooke M.E., Ramirez-GarciaLuna J.L., Rangel-Berridi K., Park H., Nazhat S.N., Weber M.H., Henderson J.E., Rosenzweig D.H. (2020). 3D Printed Polyurethane Scaffolds for the Repair of Bone Defects. Front. Bioeng. Biotechnol..

[23] Abdul-Jabbar A., Takemoto S., Weber M.H., Hu S.S., Mummaneni P.V., Deviren V., Ames C.P., Chou D., Weinstein P.R., Burch S. (2012). Surgical Site Infection in Spinal Surgery. Spine.

[24] Al Farii H., Slawaska-Eng D., Pankovitch S., Navarro-Ramirez R., Weber M. (2021). Gram-Negative Surgical Site Infections After 989 Spinal Fusion Procedures: Associated Factors and the Role of Gram-Negative Prophylactic Antibiotic Coverage. Int. J. Spine Surg..

[25] Salkind A.R., Rao K.C. (2011). Antiobiotic prophylaxis to prevent surgical site infections. Am. Fam. Physician.

[26] Lacombe J.-G., Cooke M.E., Park H., Alshammari S.M., Gawri R., Nazhat S.N., Martineau P.A., Rosenzweig D.H. (2023). Primary Human Ligament Fibroblast Adhesion and Growth on 3D-Printed Scaffolds for Tissue Engineering Applications. Surgeries.

[27] Pitaru A.A., Lacombe J.-G., Cooke M.E., Beckman L., Steffen T., Weber M.H., Martineau P.A., Rosenzweig D.H. (2020). 3D Printing to Microfabricate Stiff and Elastic Scaffolds that Mimic Ligament Tissue. Micromachines.

[28] You J., Preen R.J., Bull L., Greenman J., Ieropoulos I. (2017). 3D printed components of microbial fuel cells: Towards monolithic microbial fuel cell fabrication using additive layer manufacturing. Sustain. Energy Technol. Assess..

[29] Khan Z., He H., Chen X., Sydänheimo L., Ukkonen L., Virkki J. (2021). Testing the effects of fabrication parameters on the post-fabrication shape change of a three-dimensional printed textile platform. Text. Res. J..

[30] Parrado-Agudelo J.Z., Narváez-Tovar C. (2019). Mechanical characterization of polylactic acid, polycaprolactone and Lay-Fomm 40 parts manufactured by fused deposition modeling, as a function of the printing parameters. Iteckne.

[31] Talalwa L., Natour G., Bauer A., Drzezga A., Beer S. (2020). Radiological characteristics of a new experimental rubber elastomeric polymer used in three-dimensional printing with different infill densities and patterns. J. Phys. Commun..

[32] Stebbins N.D., Ouimet M.A., Uhrich K.E. (2014). Antibiotic-containing polymers for localized, sustained drug delivery. Adv. Drug Deliv. Rev..

[33] Palza H., Barraza B., Olate-Moya F. (2022). An Overview for the Design of Antimicrobial Polymers: From Standard Antibiotic-Release Systems to Topographical and Smart Materials. Annu. Rev. Mater. Res..

[34] Lewis G., Janna S. (2009). Estimation of the optimum loading of an antibiotic powder in an acrylic bone cement: Gentamicin sulfate in SmartSet HV. Acta Orthop..

[35] Klekamp J., Dawson J.M., Haas D.W., DeBoer D., Christie M. (1999). The use of vancomycin and tobramycin in acrylic bone cement. J. Arthroplast..

[36] Basak P., Adhikari B., Banerjee I., Maiti T.K. (2008). Sustained release of antibiotic from polyurethane coated implant materials. J. Mater. Sci. Mater. Med..

[37] Schierholz J.M., Steinhauser H., Rump A.F.E., Berkels R., Pulverer G. (1997). Controlled release of antibiotics from biomedical polyurethanes: Morphological and structural features. Biomaterials.

[38] Jansen B., Schareina S., Treitz U., Peters G., Schumacher-Perdreau F., Pulverer G. (1990). Antibiotic-Containing Polyurethanes for the Prevention of Foreign-Body Infections. Progress in Biomedical Polymers.

[39] Salimi S., Wu Y., Barreiros M.I.E., Natfji A.A., Khaled S., Wildman R., Hart L.R., Greco F., Clark E.A., Roberts C.J. (2020). A 3D printed drug delivery implant formed from a dynamic supramolecular polyurethane formulation. Polym. Chem..

[40] Fairag R., Li L., Ramirez-GarciaLuna J.L., Taylor M.S., Gaerke B., Weber M.H., Rosenzweig D.H., Haglund L. (2021). A Composite Lactide-Mineral 3D-Printed Scaffold for Bone Repair and Regeneration. Front. Cell Dev. Biol..

[41] Inzana J.A., Trombetta R.P., Schwarz E.M., Kates S.L., Awad H.A. (2015). 3D printed bioceramics for dual antibiotic delivery to treat implant-associated bone infection. Eur. Cells Mater..

[42] Lee J.-H., Baik J.-M., Yu Y.-S., Kim J.H., Ahn C.B., Son K.H., Kim J.-H., Choi E.S., Lee J.W. (2020). Development of a heat labile antibiotic eluting 3D printed scaffold for the treatment of osteomyelitis. Sci. Rep..

[43] Chen J.-R., Chen J.-R., Su C.-K. (2022). Solution Foaming–Treated 3D-Printed monolithic packing for enhanced solid phase extraction of trace metals. Talanta.

[44] Tan M.L., Zhang M., Li F., Maya F., Breadmore M.C. (2019). A three-dimensional printed electromembrane extraction device for capillary electrophoresis. J. Chromatogr. A.

[45] Kalsoom U., Hasan C.K., Tedone L., Desire C., Li F., Breadmore M.C., Nesterenko P.N., Paull B. (2018). Low-Cost Passive Sampling Device with Integrated Porous Membrane Produced Using Multimaterial 3D Printing. Anal. Chem..

[46] Su C.-K., Lin J.-Y. (2020). 3D-Printed Column with Porous Monolithic Packing for Online Solid-Phase Extraction of Multiple Trace Metals in Environmental Water Samples. Anal. Chem..

[47] Li F., Smejkal P., Macdonald N.P., Guijt R.M., Breadmore M.C. (2017). One-Step Fabrication of a Microfluidic Device with an Integrated Membrane and Embedded Reagents by Multimaterial 3D Printing. Anal. Chem..

[48] Flannagan R.S., Heit B., Heinrichs D.E. (2016). Intracellular replication of *Staphylococcus aureus* in mature phagolysosomes in macrophages precedes host cell death, and bacterial escape and dissemination. Cell. Microbiol..

[49] Kurow O., Nuwayhid R., Stock P., Steinert M., Langer S., Krämer S., Metelmann I.B. (2023). Organotypic 3D Co-Culture of Human Pleura as a Novel In Vitro Model of *Staphylococcus aureus* Infection and Biofilm Development. Bioengineering.

[50] Ning E., Turnbull G., Clarke J., Picard F., Riches P., Vendrell M., Graham D., Wark A.W., Faulds K., Shu W. (2019). 3D bioprinting of mature bacterial biofilms for antimicrobial resistance drug testing. Biofabrication.

[51] Yeh Y.-W., Huang C.-C., Kuo W.-S., Liao T.-L., Tsai T.-L., Wu P.-C. (2023). Multifunctional Hydrogel Dressing That Carries Three Antibiotics Simultaneously and Enables Real-Time Ultrasound Bacterial Colony Detection. ACS Omega.

[52] Nie B.e., Huo S., Qu X., Guo J., Liu X., Hong Q., Wang Y., Yang J., Yue B. (2022). Bone infection site targeting nanoparticle-antibiotics delivery vehicle to enhance treatment efficacy of orthopedic implant related infection. Bioact. Mater..

[53] Williams D.L., Kawaguchi B., Taylor N.B., Allyn G., Badham M.A., Rogers J.C., Peterson B.R., Sebahar P.R., Haussener T.J., Reddy H.R.K. (2020). In vivo efficacy of a unique first-in-class antibiofilm antibiotic for biofilm-related wound infections caused by *Acinetobacter baumannii*. Biofilm.

